# Age‐related differences in postsynaptic increases in sweating and skin blood flow postexercise

**DOI:** 10.14814/phy2.12078

**Published:** 2014-07-17

**Authors:** Jill M. Stapleton, Naoto Fujii, Ryan McGinn, Katherine McDonald, Glen P. Kenny

**Affiliations:** 1 Human and Environmental Physiology Research Unit, University of Ottawa, Ottawa, Ontario, Canada

**Keywords:** dose response, exercise, heat loss, nonthermal factors, skin perfusion, sweat rate

## Abstract

The influence of peripheral factors on the control of heat loss responses (i.e., sweating and skin blood flow) in the postexercise period remains unknown in young and older adults. Therefore, in eight young (22 ± 3 years) and eight older (65 ± 3 years) males, we examined dose‐dependent responses to the administration of acetylcholine (ACh) and methacholine (MCh) for sweating (ventilated capsule), as well as to ACh and sodium nitroprusside (SNP) for cutaneous vascular conductance (CVC, laser‐Doppler flowmetry, % of max). In order to assess if peripheral factors are involved in the modulation of thermoeffector activity postexercise, pharmacological agonists were perfused via intradermal microdialysis on two separate days: (1) at rest (**DOSE**) and (2) following a 30‐min bout of exercise (**Ex+DOSE**). No differences in sweat rate between the DOSE and Ex+DOSE conditions at either ACh or MCh were observed for the young (ACh: *P *=**0.992 and MCh: *P *=**0.710) or older (ACh: *P *=**0.775 and MCh: *P *=**0.738) adults. Similarly, CVC was not different between the DOSE and Ex+DOSE conditions for the young (ACh: *P *=**0.123 and SNP:* P *=**0.893) or older (ACh: *P *=**0.113 and SNP:* P *=**0.068) adults. Older adults had a lower sweating response for both the DOSE (ACh: *P *=**0.049 and MCh: *P *=**0.006) and Ex+DOSE (ACh: *P *=**0.050 and MCh: *P *=**0.029) conditions compared to their younger counterparts. These findings suggest that peripheral factors do not modulate postexercise sweating and skin blood flow in both young and older adults. Additionally, sweat gland function is impaired in older adults, albeit the impairments were not exacerbated during postexercise recovery.

## Abstract (precis)

Centrally mediated factors of nonthermal origin modulate the control of heat loss following exercise in young adults. It remains unclear if peripheral factors also play a role and whether the mechanisms modulating the postexercise suppression of heat loss differ as a function of age. We show that peripheral factors do not modulate postexercise sweating and skin blood flow in both young and older adults.

## Introduction

Thermoregulatory control of sweating and skin blood flow during postexercise recovery is altered such that at the cessation of dynamic exercise, there is a rapid decrease in sweating and skin blood flow despite a significant residual heat load (Kenny and Jay 2013). As a result, the rate of whole‐body heat loss is reduced and consequently is paralleled by a prolonged elevation in core and muscle temperatures above preexercise baseline levels for 60–90 min (Kenny et al. 2006, 2007; Kenny and Gagnon 2010). It has been suggested that centrally mediated factors of nonthermal origin (i.e., baroreceptor loading status) can modulate the control of heat loss following exercise in young adults (Carter et al. 2002; Jackson and Kenny 2003; Wilson et al. 2004; Journeay et al. 2006; Kenny et al. 2006, 2008; Jay et al. 2007). However, it has yet to be determined whether or not peripheral factors, such as sensitivity of the effector organ (i.e., sweat glands and/or skin vessels), contribute to the control of heat loss postexercise.

Peripheral factors modulating heat loss postexercise can be assessed by examining changes in sweat production and skin vasodilation to increasing doses of pharmacological agonists. For example, exogenously administering incremental doses of acetylcholine (**ACh**) and methacholine (**MCh**) can be employed to examine sweat gland function (Kenney and Fowler 1988; Inoue et al. 1999; Lee and Mack 2006; Kimura et al. 2007; Gagnon et al. 2013; Smith et al. 2013; Metzler‐Wilson et al. 2014). Furthermore, differences in sweat rate observed between ACh (hydrolyzed by acetylcholinesterase, **AChE**) and MCh (resistant to AChE) can allude to whether or not the response is mediated by AChE enzyme activity (Kimura et al. 2007). Likewise, perfusion of endothelium‐dependent (ACh) and/or endothelium‐independent (sodium nitroprusside, **SNP**) agonists in an incremental manner can be utilized to examine skin vascular function (Lee and Mack 2006; Medow et al. 2008; Bruning et al. 2012; Gagnon et al. 2013; Smith et al. 2013). To the best of our knowledge, no study has examined if peripheral mechanisms contribute to the disturbance of postexercise heat loss responses of sweating and skin blood flow.

To date, much of our limited understanding of the underlying mechanisms governing the control of postexercise heat loss responses is based on findings obtained in young adults. Human aging is associated with attenuated sweating and skin vasodilation during exercise (Anderson and Kenney 1987; Kenney and Anderson 1988; Tankersley et al. 1991; Inoue et al. 1999; Larose et al. 2013a,b,c). However, the extent to which these age‐related impairments in the thermoeffector activity may influence heat dissipation during the postexercise recovery period remains unclear. Some insight may be gleaned from a recent study by Larose et al. (2013c) who examined local and whole‐body heat loss responses in young and older adults during intermittent exercise in the heat (Larose et al. 2013c). They found that despite greater heat storage during each of the four 15‐min exercise bouts in the older adults, the magnitude of the postexercise suppression in whole‐body evaporative heat loss, as measured by direct calorimetry, was similar between the young and older males (Larose et al. 2013c). A similar pattern was measured for the local responses of sweating and skin blood flow. These findings suggest the likely possibility that the underlying factors affecting postexercise heat dissipation may be of similar origin for young and older adults.

Previous studies have compared sweat rates and skin vasodilation between young and older adults at rest with the use of pharmacological stimulation and have yielded conflicting results. While some studies have reported an attenuated sweating response in older adults as assessed by a subcutaneous injection of 5 mmol/L of MCh (Kenney 1988; Inoue et al. 1999), others observed no differences using intradermal microdialysis to administer increasing doses (1 × 10^−7^ to 0.1 mol/L) of ACh (Smith et al. 2013). Furthermore, Bruning et al. (2012) reported an attenuated ACh‐induced skin vasodilation in middle‐aged (53 ± 1 years) compared with younger (23 ± 1years) adults at the highest concentration employed (0.1 mol/L), while infusing ACh via intradermal microdialysis in a dose–response manner (Bruning et al. 2012). Others, however, have not observed any differences in skin blood flow between young and older adults receiving doses of ACh from 1 × 10^−7^ to 0.1 mol/L (Holowatz et al. 2005; Smith et al. 2013). To date, no study has evaluated if age‐related differences in sweating or skin vasodilation exist at higher doses of ACh or MCh (>0.1 mol/L). It remains to be determined if older adults have an attenuated responsiveness to the administration of pharmacological agonists (>0.1 mol/L) compared to their younger counterparts and whether or not the same pattern of response exists postexercise.

Thus, the purpose of this study was twofold: to examine (1) the extent to which peripheral factors (i.e., sweat gland and skin vasodilatory function) contribute to the postexercise suppression of heat loss responses; and (2) whether there are differences in the mechanisms modulating postexercise heat loss as a function of age. We hypothesized that: (1) peripheral factors would not modulate the postexercise suppression of heat loss as determined by local measurements of sweating and skin blood flow and, (2) the mechanisms for postexercise suppression of heat loss would not differ as a function of age, but older adults would have an attenuated responsiveness to the administration of the pharmacological agonists compared to their younger counterparts.

## Methods

### Ethical approval

This study was approved by the University of Ottawa Health Sciences and Science Research Ethics Boards, in accordance with the Declaration of Helsinki. Written, informed consent was obtained from all the participants prior to their involvement in the study.

### Participants

Sixteen males volunteered for the study and were divided into two groups of eight young (18–25 years) and eight older (61–70 years) adults. All participants were healthy, nonsmoking, physically active males free from cardiovascular disease and diabetes. Physical characteristics of the participants are presented in Table 1.

**Table 1.** Participant characteristics for young and older adults

Group	Age (years)	Height (m)	Body mass (kg)	Body surface area (m^2^)	Body fat (%)	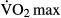 (mL/kg/min)
Young	22 ± 3*	1.78 ± 0.10	81.7 ± 7.7	1.99 ± 0.11	14.3 ± 3.5*	46.3 ± 3.5*
Older	65 ± 3	1.75 ± 0.05	77.6 ± 12.9	1.94 ± 0.17	21.6 ± 6.7	34.3 ± 8.3

Values are mean ± standard deviation. 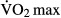, rate of maximum oxygen consumption. A significant difference (*P *≤**0.05) between young and older adults is denoted by an asterisk (*).

### Experimental procedures

Each participant completed one preliminary and two experimental sessions. The experimental sessions were performed in a random order and on separate days with a minimum of 72 h and maximum of 2 weeks between sessions. During the preliminary session, body height, mass, and density, as well as maximum oxygen uptake (VO_2_max) were determined. Body height was determined using a stadiometer (Detecto, model 2391, Webb City, MO), whereas body mass was measured using a digital high‐performance weighing terminal (model CBU150X; Mettler Toledo Inc., Mississauga, ON, Canada). Body surface area was subsequently calculated from the measurements of body height and mass (DuBois and DuBois 1916). Body density was measured using the hydrostatic weighing technique, and body fat percentage was calculated using the Siri equation (Siri 1956). VO_2_max was measured during a progressive cycle ergometer protocol which consisted of a 2‐min warm‐up at 40 W followed by 20 W increments every minute until the participant could no longer maintain a pedaling cadence of at least 60 rpm. Continuous electrocardiographic monitoring was used for the older males during the maximal exercise test under the supervision of a qualified technician.

Participants performed the experimental sessions at the same time of day and were asked to drink 500 mL of water the night prior to, as well as the morning of the experimental session. They were also asked to refrain from alcohol, caffeine, and exercise 24 h prior to experimentation. Upon arrival at the laboratory, the participants provided a urine sample and a baseline body mass was measured. They subsequently rested quietly in an upright semirecumbent posture on a bed in a room set to an ambient temperature of 24°C and 20% relative humidity. During this time, three microdialysis fibers (MD 2000; Bioanalytical Systems, West Lafayette, IN) were placed in the dermal space of the forearm under aseptic conditions. To place the fibers, a 25‐gauge needle was inserted into the dermal space of the lateral mid‐anterior aspect of the left forearm and then exited the skin 20–25 mm away from the point of entry. The microdialysis fiber was inserted through the lumen of the needle. The needle was subsequently withdrawn, leaving the semipermeable membrane (30 KDa cutoff, 10 mm membrane) in place under the skin. After insertion, the fibers were perfused with lactated Ringer's solution at a rate of 2 *μ*L/min via a perfusion pump (CMA/400; CMA Microdialysis, Solna, Sweden).

For one of the experimental conditions (**DOSE**), participants remained resting in an upright semirecumbent posture on the bed in a nonheat stress environment (i.e., ambient temperature of 24°C and 20% relative humidity) for 60–90 min after the fiber placement (to allow for hyperemia associated with fiber insertion trauma to subside; Anderson et al. 1994). Baseline resting data were obtained for 10 min following the hyperemia response. Subsequently, increasing doses of **MCh (***site 1*) and **ACh** (*site 2*) were infused in a dose‐dependent manner at two mid‐anterior forearm skin sites to assess the sweating response. The ACh infusion at site 2 as well as infusion of **SNP** (*site 3*) was used in a dose‐dependent manner to assess skin vasodilation (all pharmacological agonists were from Sigma Aldrich, Oakville, ON, Canada). All pharmacological agonists were infused in 10‐fold increments, from 1 × 10^−6^ to 1 mol/L for MCh and ACh, and from 5 × 10^−6^ to 5 × 10^−2^ mol/L for SNP (Gagnon et al. 2013). Each dose was initially primed through the microdialysis membrane at an infusion rate of 100 *μ*L/min for ~1 min, thereafter, each dose was infused for 8 min at a rate of 2 *μ*L/min. This amount of time ensured a plateau in sweat rate or skin blood flow was reached at each concentration of the agonists. A higher dose of ACh and MCh (1.5 mol/L) was infused at the end for an additional 25 min, while the maximum dose of SNP (5 × 10^−2^ mol/L) continued to be infused to ensure a steady‐state maximal response to the highest concentration employed.

For the second experimental condition (**Ex+DOSE**), after fiber placement, the participants entered a thermal chamber regulated to an ambient air temperature of 30°C and 20% relative humidity. The participants rested for 60–90 min on a semirecumbent cycle ergometer (Corival; Lode B.V., Groningen, Netherlands) while the remainder of the instrumentation was placed. Once the instrumentation was placed and the hyperemia response had subsided, baseline resting data were obtained for 10 min after which the participants performed a 30‐min exercise bout. To ensure that both groups received a similar heat load, they exercised at the same constant rate of metabolic heat production of ~250 W (equivalent to 46.3 ± 4.7% VO_2_max for the young and 55.7 ± 6.9% VO_2_max for the older males). Following the exercise bout, participants rested for 15 min to allow local sweat rate and skin blood flow to return to baseline resting values (Kenny et al. 2007). At this point, the dose–response relationships for sweating and skin vasodilation were assessed in the same manner as for the DOSE experimental session.

### Measurements

The ventilated capsule technique was employed for the purpose of measuring local sweat rate. Sweat rate was measured from 3.8‐cm^2^ plastic capsules attached to the skin with adhesive rings and topical skin glue (Collodion HV; Mavidon Medical products, Lake Worth, FL). The sweat capsules were placed directly over the fiber membrane of each agonist sites (i.e., skin sites 1 and 2). The sweat capsule at the ACh site (*site 2*) also housed the laser‐Doppler flow probe (see details below), allowing for the simultaneous measurement of local sweat rate and skin blood flow. Compressed dry air was passed through each capsule at a rate of 0.5 L/min. Long tubes were used to supply the dry gas to and from the ventilated capsules to ensure optimal equilibration with ambient environmental conditions for the experimental trial. Water content of the effluent air was measured using high precision dew point mirrors (model 473; RH systems, Albuquerque, NM) or capacitance hygrometers (Vaisala, Woburn, WA). Both instruments offer precise, quality measures of changes in humidity at the skin (RH systems: dew point accuracy = ±0.2°C and Viasala: absolute humidity accuracy = ~1.08 g/m^3^). Local sweat rate was calculated using the difference in water content between effluent and influent air multiplied by the flow rate and normalized for the skin surface area under the capsule. Local skin blood flow was estimated at 32 Hz using laser‐Doppler velocimetry (PeriFlux System 5000; Perimed AB, Stockholm, Sweden). A laser‐Doppler probe (integrating probe 413; Perimed AB) was placed directly over the microdialysis membrane at the ACh (*site 2*) and SNP (*site 3*) sites. Cutaneous vascular conductance (CVC) was subsequently calculated as the ratio of skin blood flow perfusion units to mean arterial pressure and expressed as a percentage of maximum.

Systolic and diastolic blood pressures were determined manually using brachial auscultation at the end of each 8‐min infusion during the DOSE condition. Mean arterial pressure was then calculated as diastolic blood pressure + 1/3 × pulse pressure (difference between systolic and diastolic pressure). Additionally, mean arterial pressure was measured continuously using a Finometer (Finapres Medical Systems, Amsterdam, the Netherlands) from the beat‐to‐beat recording of the left middle finger arterial pressure waveform with the volume‐clamp method (Penaz 1973) and physiocal criteria (Wesseling et al. 1995) during the Ex+DOSE condition. The left middle finger was supported at heart level for calibration and for the duration of the experimental protocol. Blood pressures were verified during the Ex+DOSE condition by manual brachial auscultation.

Rectal temperature was measured during the Ex+DOSE condition using a general purpose thermocouple temperature probe (Mallinckrodt Medical Inc., St‐Louis, MO) inserted to a minimum of 12 cm past the anal sphincter. Rectal temperature data were collected using a HP Agilent data acquisition module (model 3497A; Agilent Technologies Canada Inc., Mississauga, ON, Canada) at a rate of one sample every 15 sec and simultaneously displayed and recorded in spreadsheet format on a personal computer with LabVIEW software (Version 7.0; National Instruments, Austin, TX).

A preexperimental test of urine sample was obtained to ensure all participants were in a euhydrated state. Urine‐specific gravity was determined in duplicate using a handheld total solids refractometer (model TS400; Reichter Inc., Depew, NY).

### Data analysis

To determine the concentration of the agonist causing 50% of the maximal response (EC_50_), dose–response curves were created by plotting local sweat rate and CVC as a function of the log concentration of the agonist and fitted using a nonlinear regression analysis with a Hill slope of 1 (GraphPad Prism 6.0; GraphPad Software, La Jolla, CA; Davis et al. 2007; Kimura et al. 2007). The log EC_50_ is an indicator of sensitivity of the end organ to the agonist, where a negative log EC_50_ closer to 0 indicates a lower sensitivity. Baseline resting data for both the DOSE and Ex+DOSE conditions were obtained by averaging the final 5 min of the 10‐min baseline resting period. Postexercise data were obtained for the Ex+DOSE condition only by averaging the final min of the 15‐min postexercise recovery period. Sweat rate and CVC averages were obtained during the final min of each 8‐min dose. The dose–response curves were compared within age groups for the DOSE and Ex+DOSE conditions to assess whether peripheral factors influence heat loss responses postexercise. Additionally, the dose–response curves were compared between age groups for the DOSE and Ex+DOSE conditions separately to assess the effect of age on end‐organ function and on the mechanism of the postexercise suppression of heat loss responses.

### Statistical analysis

Sweating, CVC, mean arterial pressure, and rectal temperature (Ex+DOSE only) data were analyzed using a two‐way repeated measures analyses of variance protocol using the repeated factor of agonist concentration (eight levels: 10‐fold increments from 1 × 10^−6^ to 1 and 1.5 mol/L for MCh and ACh and from 5 × 10^−6^ to 5 × 10^−2^ mol/L for SNP) and the nonrepeated factor of test condition (DOSE vs. Ex+DOSE) and age (two levels: young and older), separately. When a significant main effect was observed for test condition (DOSE vs. Ex+DOSE), post hoc comparisons were carried out using Student's paired two‐tailed *t‐*tests. Likewise, when a significant main effect was observed for age (young vs. older), post hoc comparisons were carried out using Student's unpaired two‐tailed *t‐*tests. Additionally, physical characteristics as well as baseline resting, end exercise (Ex+DOSE only) and 15‐min postexercise (Ex+DOSE only) values, the log EC_50_ and absolute maximal CVC values were analyzed using Student's unpaired two‐tailed *t‐*tests. Within group comparisons (i.e., baseline resting vs. 15‐min postexercise values) were analyzed using Student's paired two‐tailed *t‐*tests. The level of significance for all analyses was set at *P *≤**0.05. Analyses were performed using commercially available statistical software (GraphPad Prism 6.0; GraphPad Software, La Jolla, CA). All values are reported as mean ± standard deviation unless otherwise indicated as standard error.

## Results

### Participant characteristics

Participant characteristics are presented in Table 1. There were no differences in height (*P *=**0.460), body mass (*P *=**0.459), and body surface area (*P *=**0.534) between groups. However, the younger males had a greater maximum oxygen consumption relative to body mass (*P *<**0.001). On the day of both experimental sessions, baseline urine‐specific gravity did not significantly differ between groups (DOSE: young = 1.026 ± 0.006 vs. older = 1.019 ± 0.005, *P *=**0.229 and Ex+DOSE: young = 1.017 ± 0.007 vs. older = 1.017 ± 0.004, *P *=0.967). During the Ex+DOSE condition, young and older adults exercised at a fixed rate of heat production which was kept similar between the young (257 ± 12 W/m^2^) and older (240 ± 18 W/m^2^, *P *=**0.114) adults.

### Postsynaptic sweating during no‐exercise resting (DOSE) and postexercise recovery (Ex+DOSE)

**Figure 1.**
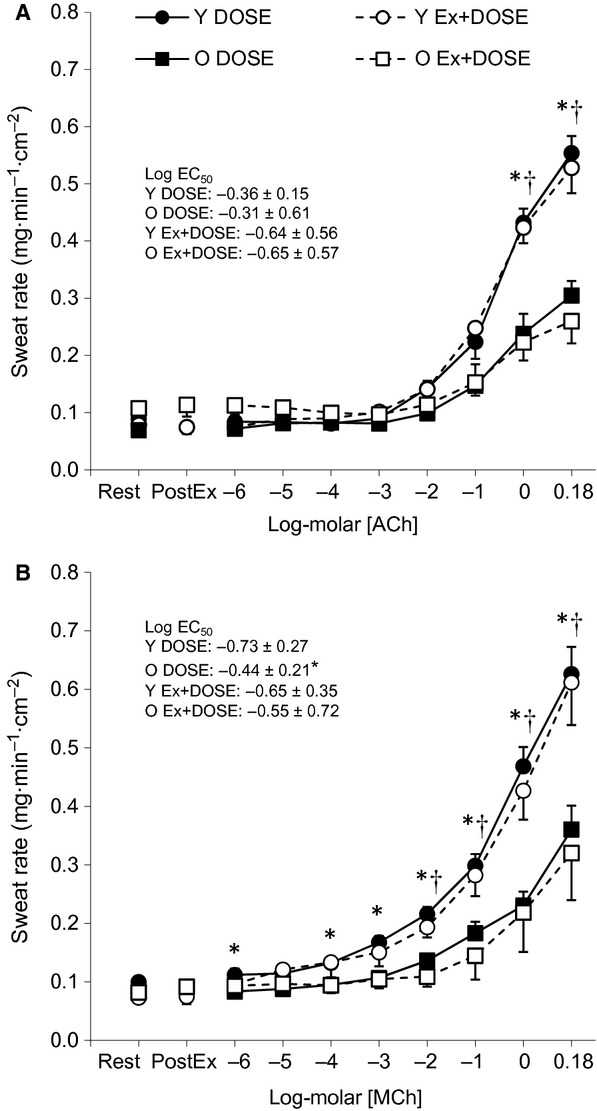
Mean ± standard error values for the DOSE (closed symbols) and Ex+DOSE (open symbols) condition for sweat rate to incremental doses of acetylcholine (ACh, Panel A) and methacholine (MCh, Panel B) in young (Y, circles) and older (O, squares) adults. Data are presented during baseline rest, 15‐min postexercise (PostEx), and during the plateau phase of each dose. *Significant difference between young and older adults for the DOSE condition. ^†^Significant difference between young and older adults for the Ex+DOSE condition (*P *≤**0.05).

Sweating responses at baseline rest, 15‐min postexercise (Ex+DOSE only) and to incremental doses of ACh and MCh for the young and older adults are presented in Figure 1A and B, respectively. Baseline resting sweat rate was similar between the DOSE and Ex+DOSE conditions in both young and older adults for the ACh (young: *P *=**0.614, older: *P *=**0.105) and MCh (young: *P *=**0.123, older: *P *=**0.666) skin sites. For the Ex+DOSE condition, there were no differences in sweat rates between baseline rest and 15‐min postexercise in either young or older adults for both the ACh (young: *P *=**0.453, older: *P *=0.301) and MCh (young: *P *=**0.348, older: *P *=**0.152) sites. Likewise, there was no main effect of the experimental condition such that sweating responses were similar between DOSE and Ex+DOSE in young and older adults during the incremental doses of both ACh (young: *P *=**0.992, older: *P *=**0.775) and MCh (young: *P *=**0.710, older: *P *=**0.738).

### Age‐related effects on postsynaptic sweating

#### No‐exercise resting condition (**DOSE**)

There were no significant differences in baseline resting sweat rate between young and older males at both the ACh (*P *=**0.230) and MCh (*P *=**0.276) skin sites. Sweating increased as a function of increasing concentrations for both ACh and MCh (both *P *≤**0.001). Furthermore, there was a main effect of age on local sweat rate for ACh (*P *=**0.049) and MCh (*P *=**0.006). For ACh, sweat rate did not differ between age groups at the lower concentrations (i.e., 1 × 10^−6^–1 × 10^−1^ mol/L), but was greater in the young compared to the older males at the two higher concentrations (1 and 1.5 mol/L) employed (both *P *<**0.05). For MCh, sweat rate was greater in the young compared to the older males at 1 × 10^−6^ and 1 × 10^−4^ to 1.5 mol/L (all *P *<**0.05). The log EC_50_ did not differ between age groups for ACh (*P *=**0.814), but was lower (i.e., further away from 0) for the young compared to the older adults for MCh (*P = *0.035).

#### Postexercise resting recovery condition (**Ex+DOSE**)

There were no significant differences in baseline resting sweat rate between the young and older adults at both the ACh (*P *=**0.351) and MCh sites (*P *=**0.934). Likewise, there were no differences observed between groups in sweat rate 15‐min postexercise for ACh (*P *=**0.121) or MCh (*P *=**0.246). Sweating increased as a function of increasing concentrations of both ACh and MCh (both *P *<**0.001). There was a main effect of age on local sweat rate for ACh (*P *=**0.05) and MCh (*P *=**0.029). For ACh, sweat rate did not differ between age groups at the lower concentrations (i.e., 1 × 10^−6^ to 1 × 10^−1^ mol/L), but was greater in young compared to older males at the two highest concentrations (1 and 1.5 mol/L) employed (both *P *<**0.05). For MCh, sweat rate was greater in young compared to older males at 1 × 10^−2^ to 1.5 mol/L (all *P *<**0.05). However, the log EC_50_ did not differ between age groups for ACh (*P *=**0.483) or MCh (*P* = 0.362).

### Postsynaptic skin vasodilation during no‐exercise resting (DOSE) and postexercise recovery (Ex+DOSE)

**Figure 2.**
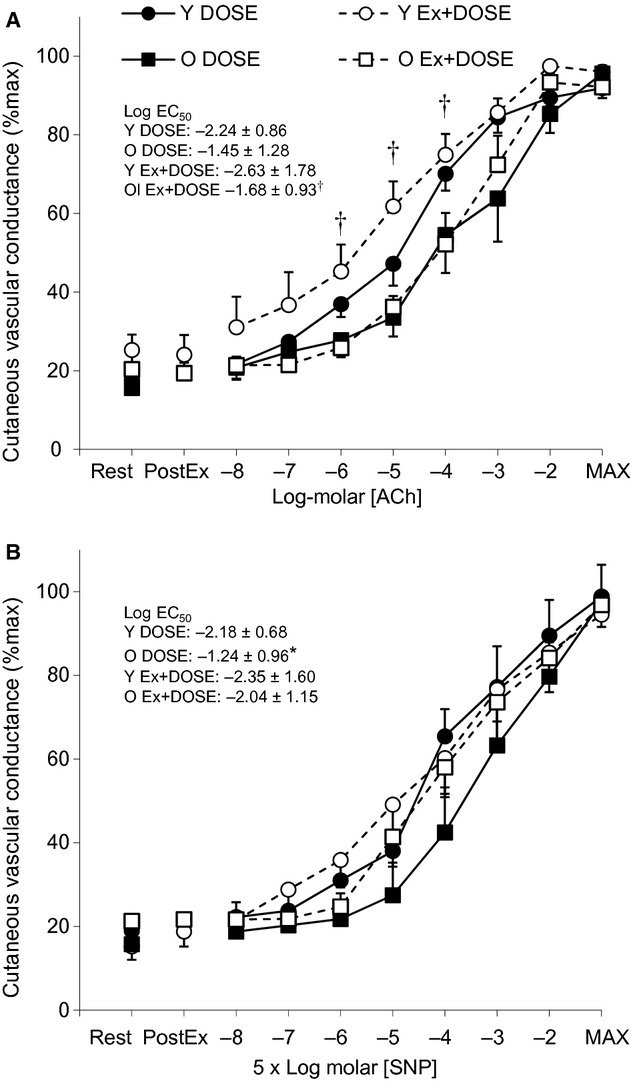
Mean ± standard error values for the DOSE (closed symbols) and Ex+DOSE (open symbols) conditions for cutaneous vascular conductance to incremental doses of acetylcholine (ACh, Panel A) and sodium nitroprusside (SNP, Panel B) in young (Y, circles) and older (O, squares) adults. Data are presented during baseline rest, 15‐min postexercise (PostEx), and during the plateau phase of each dose. *Significant between young and older adults for the DOSE condition. ^†^Significant between young and older adults for the Ex+DOSE condition (*P *≤**0.05).

Cutaneous vascular conductance responses at baseline rest, 15‐min postexercise (Ex+DOSE only) and to incremental doses of ACh and SNP for the young and older adults are presented in Figure 2A and B, respectively. Baseline resting values for CVC were similar between young and older adults for the DOSE and Ex+DOSE conditions for both ACh (young: *P *=**0.308, older: *P *=**0.113) and SNP (young: *P *=**0.949, older: *P *=**0.068) skin sites. For the Ex+DOSE condition, CVC returned to preexercise baseline levels such that there was no difference between baseline rest and 15‐min postexercise levels in young and older adults for both the ACh (young: *P *=**0.826, older: *P *=**0.853) and SNP (young: *P *=**0.187, older: *P *=**0.883) sites. Additionally, CVC responses were not different in either young or older adults at the ACh (young: *P *=**0.123, older: *P *=**0.832) or SNP (young: *P *=**0.893, older: *P *=**0.360) sites between the DOSE and Ex+DOSE conditions during the infusion of the incremental doses.

### Age‐related effects on postsynaptic skin vasodilation

#### No‐exercise resting condition (**DOSE**)

There were no significant differences in baseline resting CVC between young and older males at both the ACh (*P *=**0.864) and SNP (*P *=**0.507) skin sites. CVC increased as a function of increasing concentrations of both ACh and SNP (both *P *≤**0.001), but there was no main effect of age on CVC for ACh (*P *=**0.127) or SNP (*P *=**0.131). In contrast, the log EC_50_ was lower for the young compared to the older adults for SNP (*P *=**0.041), but not for ACh (*P *=**0.087). Additionally, maximal absolute CVC values did not differ between young (ACh: 1.65 ± 0.46 and SNP: 1.78 ± 0.48 perfusion units/mmHg) and older (ACh: 1.78 ± 0.83 and SNP: 1.97 ± 0.62 perfusion units/mmHg) adults (both *P > *0.10). Mean arterial pressure did not change throughout the protocol (*P = *0.120) and there was no main effect of age (*P = *0.873) between young (average: 87 ± 10 mmHg) and older (average: 87 ± 8 mmHg) adults.

#### Postexercise resting recovery condition (**Ex+DOSE**)

There were no significant differences in preexercise baseline resting CVC between the young and older males at both the ACh (*P *=**0.401) or SNP (*P *=**0.191) skin sites. Likewise, there were no differences between groups observed in CVC 15‐min postexercise at the ACh (*P *=**0.425) or SNP (*P *=**0.530) sites. CVC increased as a function of increasing concentrations of both ACh and SNP (both *P *≤**0.001). There was a main effect of age on CVC for ACh (*P *=**0.014). CVC was greater in the young at 1 × 10^−4^ to 1 × 10^−2^ mol/L compared to the older males (all *P *<**0.05). In contrast, there was no main effect of age on CVC for SNP (*P *=**0.573). Consequently, the log EC_50_ was lower for the young compared to older adults for ACh (*P *=**0.044), but was not different between groups for SNP (*P *=**0.665). Additionally, maximal absolute CVC values did not differ between young (ACh: 1.91 ± 0.53 and SNP: 1.37 ± 0.70 perfusion units/mmHg) and older (ACh: 1.63 ± 0.85 and SNP: 1.23 ± 0.44 perfusion units/mmHg) adults (both *P > *0.10).

### Age‐related effects on rectal temperature and mean arterial pressure responses

There were no differences between age groups in baseline resting (*P *=**0.835), end‐exercise (*P *=**0.572), or 15‐min postexercise (*P *=**0.933) rectal temperatures (Table 2). Additionally, rectal temperature decreased as a function of time from end of exercise to end of the dose–response protocol (*P *<**0.001), but was not different between age groups (*P *=**0.354, Table 2). However, the older adults had a greater change in rectal temperature from preexercise baseline resting values at the end of the dose–response protocol (0.50 ± 0.28°C) compared to the young adults (0.23 ± 0.13°C, *P = *0.041). There were no age group differences at baseline resting (*P *=**0.336), end exercise (*P *=**0.253), or 15‐min postexercise in mean arterial pressure (*P *=**0.551, Table 2). However, the older adults had a significantly lower mean arterial pressure at 15‐min postexercise compared to preexercise baseline resting values (Table 2, *P* *=* 0.049). No differences were observed in the young adults (*P = *0.369). Mean arterial pressure did not differ over time during the agonist infusion protocol (*P *>**0.10) and was not different between age groups (*P *=**0.217, Table 2).

**Table 2.** Mean arterial pressure and rectal temperature responses for Ex+DOSE during baseline, end of exercise (End‐Ex), following 15 min of recovery (Post‐Ex), and during the plateau phase for each dose for young and older adults

	Baseline	End‐Ex	Post‐Ex	Dose 1	Dose 2	Dose 3	Dose 4	Dose 5	Dose 6	Dose 7	Dose 8
MAP											
Young	85 ± 6	99 ± 8	84 ± 8	84 ± 8	83 ± 9	84 ± 11	84 ± 9	84 ± 9	85 ± 9	85 ± 10	86 ± 8
Older	88 ± 8	103 ± 12	81 ± 8^†^	82 ± 8	84 ± 8	83 ± 8	83 ± 8	82 ± 9	81 ± 8	84 ± 8	82 ± 8
T_re_											
Young	36.87 ± 0.42	37.47 ± 0.44^†^	37.44 ± 0.45^†^	37.41 ± 0.44^†^	37.35 ± 0.43^†^	37.31 ± 0.41^†^	37.21 ± 0.42^†^	37.15 ± 0.43^†^	37.11 ± 0.42^†^	37.09 ± 0.42^†^	37.06 ± 0.39^†^
Older	36.84 ± 0.25	37.36 ± 0.28^†^	37.45 ± 0.13^†^	37.43 ± 0.16^†^	37.41 ± 0.21^†^	37.40 ± 0.22^†^	37.35 ± 0.25^†^	37.33 ± 0.23^†^	37.30 ± 0.25^†^	37.27 ± 0.23^†^	37.27 ± 0.22^†^

Values are mean ± standard deviation.

MAP, mean arterial pressure (mmHg); T_re_, rectal temperature (°C).

A significant difference (*P *≤**0.05) from baseline resting within age group is denoted by a dagger (†).

## Discussion

A key finding of this study was our observation that the dose–response relationships with incremental pharmacological agonists (ACh, MCh and SNP) were similar between the no‐exercise resting condition (DOSE) and the postexercise resting recovery period (Ex+DOSE) for both sweating and skin vasodilation. Moreover, we show that the pattern of response was similar for both the young and older adults. However, we showed that older adults had an attenuated sweating responsiveness to the administration of pharmacological muscarinic receptor agonists (ACh and MCh) compared to the young adults. This impairment was observed during both the no‐exercise resting (DOSE) and the postexercise resting recovery (Ex+DOSE) conditions. Conversely, our findings for age‐related differences in CVC are less conclusive such that CVC was lower in the older compared to young adults with the use of ACh during the Ex+DOSE condition only. Together these findings suggest that peripheral factors do not modulate the postexercise suppression of heat loss responses of sweating and CVC in both young and older adults, despite the age‐related impairments in sweat gland function.

It has been well documented that sweating and skin blood flow return to baseline levels in the first ~20 min following the cessation of dynamic exercise despite sustained elevations in core and muscle temperatures (Thoden et al. 1994; Kenny et al. 2006, 2009). Consistent with these observations, we observed a rapid reduction in sweating and CVC to baseline levels within 15 min of postexercise recovery (Figures 1 and 2) despite rectal temperature remaining significantly elevated above baseline resting values by ~0.6°C (Table 2). This postexercise disturbance in thermal homeostasis is thought to be the result of a nonthermal, centrally mediated suppression of the thermoeffector responses of sweating, and skin blood flow (Kenny and Jay 2013). This notion is supported in part by the fact that the core temperature threshold for sweating and skin vasodilation, which is thought to be determined by central drive (Nadel et al. 1974; Gisolfi and Wenger 1984), is elevated following dynamic exercise (Jackson and Kenny 2003; Kenny et al. 2003). However, no changes in thermal sensitivity, an indicator of peripheral modulation, were observed (Nadel et al. 1971, 1974; Jackson and Kenny 2003). Nonetheless, there is no direct evidence indicating the impaired heat loss responses postexercise are entirely due to central mechanisms. That is, other mechanisms of peripheral origin, such as changes in the responsiveness of the effector organ (i.e., sweat glands and/or skin vessels) to pharmacological stimuli, may also be involved in the impaired heat loss responses postexercise. In the following section, we discuss how this study findings provide important new information to address this knowledge gap.

### Postexercise sweating and CVC in young adults

In this study, we did not observe any difference in sweating with incremental doses of ACh or MCh between the DOSE and Ex+DOSE conditions in the young adults (Fig. 1A and B). This finding indicates that the cholinergic sensitivity of the muscarinic receptors on the sweat gland is unaltered by a previous bout of exercise. Alternatively, studies have shown that AChE is involved in the regulation of sweating at low‐to‐moderate levels during passive heat stress (Shibasaki and Crandall 2001). It is plausible, therefore, that the rapid postexercise suppression of sweating may be due to increased activity of the AChE enzyme. If this were true, we would expect to observe a rightward shift in the dose–response curve for ACh during the Ex+DOSE compared to the DOSE condition with minimal difference in the dose–response curve between the DOSE and Ex+DOSE conditions for MCh. However, this was not the case in this study (Fig. 1A and B). Based on our observations, it appears that the postexercise suppression of the sweating response is independent of the modulation of AChE enzyme activity. We cannot, however, eliminate its involvement early in recovery (i.e., in the first 15 min) since the dose–response protocol only commenced 15 min into postexercise recovery. Based on our results, we show that the postexercise attenuation of the sweating response is the result of a centrally mediated modulation as previously proposed (Journeay et al. 2006; Shibasaki and Crandall 2010; Kenny and Jay 2013).

ACh‐induced skin vasodilation is in part due to nitric oxide‐dependent mechanisms (Kellogg 2005; Medow et al. 2008; Bruning et al. 2012; Fujii et al. 2013). Our results demonstrate no difference in the dose–response curves for CVC between the DOSE and Ex+DOSE conditions using ACh in young adults (Fig. 2A). Furthermore, our laboratory recently found that L‐NAME, a nonselective nitric oxide inhibitor, reduced CVC relative to the control condition only during the first ~10 min into postexercise recovery (McGinn et al. 2014). Together, these results imply that peripherally modulated mechanisms of skin vasodilation including changes in cholinergic sensitivity of the muscarinic receptor on the endothelium and nitric oxide‐mediated pathways are not modified postexercise. In this study, similar to using ACh, we did not observe any differences in skin vasodilation in the young adults with incremental doses of SNP between the DOSE and Ex+DOSE conditions (Fig. 2B). Given that SNP is a nitric oxide donor that acts directly on the smooth muscle cell to cause relaxation and therefore vasodilation, we conclude that vascular smooth muscle function is also not altered postexercise. Consequently, as in the case of the observed changes in postexercise sweating, we show that postexercise control of skin vasodilation in young adults is modulated by central factors.

### Postexercise sweating and CVC in older adults

Similar to the young, the sweating response to administration of ACh and MCh in the older adults did not differ between the no‐exercise resting (DOSE) and postexercise resting recovery (Ex‐DOSE) conditions (Fig. 1A and B). Thus, we show for the first time that despite an age‐related attenuation in the sweating response (discussed below); as in the case of younger adults, the control of sweating in the postexercise recovery period in older adults is likely not mediated by mechanisms of peripheral origin. Likewise, the pattern of response in CVC was not different between the DOSE and Ex+DOSE conditions as assessed using the administration of incremental doses of ACh and SNP (Fig. 2A and B). Thus, we show that the control of postexercise skin vasodilation is most likely due to central mechanisms; a response which parallels that observed in young adults.

### Effects of aging on sweating and CVC

Numerous studies have examined age‐related differences in thermoregulatory sweating during exercise and some have found reduced local/whole‐body sweating and/or altered core temperature onset thresholds and thermosensitivity of the sweating response (Anderson and Kenney 1987; Kenney and Anderson 1988; Tankersley et al. 1991; Inoue et al. 1999; Larose et al. 2013a,b,c). In this study, older adults likely had greater residual heat storage postexercise as indicated by a greater change in rectal temperature relative to baseline resting values at the end of the dose–response protocol in the older (0.50 ± 0.28°C) compared to the young (0.23 ± 0.13°C) adults. Despite this greater amount of heat, they were not able to produce more sweat during the dose–response protocol. It has been postulated that the age‐related impairments in sweating are due to differences in end‐organ function such as cholinergic sensitivity of the muscarinic receptors on sweat glands (Kenney and Fowler 1988; Inoue et al. 1999). In this study, we found that the dose‐dependent sweating response to the administration of ACh and MCh was lower in older males relative to young males for the no‐exercise resting conditions (DOSE; Fig. 1A and B). On the contrary, a recent study by Smith et al. (2013) reported no age‐related differences in the sweating response to administration of ACh from 1 × 10^−7^ to 1 × 10^−1^ log‐molar performed during resting under nonheat stress conditions. While at first glance our results appear to contradict the findings by Smith et al. (2013), it is important to note that we only observed age‐related decreases in sweating at the two highest doses of ACh (1 and 1.5 mol/L). No significant difference in sweating between young and older males was observed at and below a concentration of ACh of 10^−1^ log‐molar (Fig. 1A). Taken together, it is plausible that a concentration of ACh > 10^−1^ log‐molar is required to clearly observe age‐related reductions in end‐organ sweat gland function. On the other hand, age‐related differences in sweating were observed with MCh even at a lower concentration (log‐molar 1 × 10^−4^). It is possible, therefore, that the effect of age on impairments in sweating can be masked or reduced by AChE enzyme activity. As such, MCh would be more suitable in the assessment of age‐related differences in cholinergic sensitivity of muscarinic receptors on sweat glands.

Previous studies examining the effect of age on ACh‐dependent skin vasodilation have yielded mixed conclusions (Holowatz et al. 2005; Bruning et al. 2012; Smith et al. 2013). One study found skin vasodilation to be impaired in older adults (Bruning et al. 2012), whereas others have reported no age‐related differences (Holowatz et al. 2005; Smith et al. 2013). Consistent with previous reports, our findings were also inconclusive. While we did not observe attenuated skin vasodilation during the DOSE condition to ACh in the older compared to young adults (Fig. 2A), we showed that endothelium function was impaired in the older adults during the Ex+DOSE condition (Fig. 2A). Given that ACh‐mediated skin vasodilation occurs via nitric oxide‐dependent mechanisms as discussed above, the age‐related reductions in skin vasodilation (Fig. 2A) may reflect age‐related decreases in nitric oxide‐dependent skin vasodilation. Supporting this concept, it has been suggested that aging lowers nitric oxide‐dependent skin vasodilation to ACh (Bruning et al. 2012).

For the first time, our study assessed skin vasodilation in response to incremental doses of SNP in young and older adults. Our results show that the log EC_50_ for CVC was greater (i.e., closer to 0) in the older adults in comparison to the young adults during the DOSE condition (Fig. 2B). This result suggests age‐related decreases in smooth muscle sensitivity/responsiveness to nitric oxide (i.e., endothelium‐independent vasodilation). This finding is consistent with one study demonstrating that expression of soluble guanylyl cyclase, the receptor for nitric oxide which causes smooth muscle relaxation, decreases with increasing age, which has been observed in the aortic ring of rats (Kloss et al. 2000). In contrast, we did not observe a difference in the log EC_50_ for CVC during the Ex+DOSE condition. Furthermore, the CVC responses to incremental doses of SNP were not significantly different between the young and older adults during the DOSE or Ex+DOSE condition. It remains unclear why the sensitivity to nitric oxide was impaired in the older adults during the DOSE condition only. Further studies are required to examine potential mechanisms.

Another interesting observation is that age‐related reductions in skin vasodilation to ACh were not observed at the higher concentrations employed in this study (Fig. 2A). This may indicate that there are other mechanisms compensating for the reduced nitric oxide‐dependent mechanisms. One possibility is that endothelium‐derived hyperpolarizing factors (EDHFs) are acting as a redundant mechanism as has been shown when nitric oxide‐dependent vasodilation is reduced in humans (Luksha et al. 2009) and rats (Goto et al. 2012). EDHFs cause relaxation of smooth muscle cells and thus vasodilation in human skin by stimulating calcium‐activated potassium (KCa) channels (Lorenzo and Minson 2007; Brunt and Minson 2012; Cracowski et al. 2013). Taken together, it is plausible that age‐related impairment of nitric oxide‐dependent mechanisms may upregulate the EDHF pathway(s). This may explain the lack of an age‐related difference in CVC at the higher doses of ACh.

Some studies have reported that maximal CVC decreases with age (Martin et al. 1995; Minson et al. 2002; Hodges et al. 2010), while others found no differences in maximal CVC induced by SNP between young and older adults (Bruning et al. 2012; Smith et al. 2013). We did not observe a reduced absolute maximal CVC induced by 50 mmol/L SNP in the older adults relative to their younger counterparts. The disparity in the pattern of response may be due to regional differences (Inoue and Shibasaki 1996), where an age‐related effect on the maximal skin vasodilatory capacity is not always evident at all areas of the skin, even when measured within the same body part (i.e., forearm).

## Conclusions

This study demonstrates that the postexercise suppression of heat loss responses is not mediated by the factors of peripheral origin. This is evidenced by our observation that no differences in sweating or CVC with incremental doses of pharmacological agonists were observed during the no‐exercise resting condition (DOSE) or the postexercise resting recovery period (DOSE+Ex) in both young and older adults. Furthermore, while we show marked impairment in sweat gland function in the older adults, the mechanisms underlying age‐related changes in CVC were less conclusive.

## Acknowledgments

The authors thank all the members of the Human and Environmental Physiology Research Unit who assisted with data collection. We would also like to thank all the participants who volunteered for this study.

## Conflict of Interest

The authors declare that they have no competing interests.
